# Energy conversion in magneto-rheological elastomers

**DOI:** 10.1080/14686996.2017.1377590

**Published:** 2017-10-13

**Authors:** Gael Sebald, Masami Nakano, Mickaël Lallart, Tongfei Tian, Gildas Diguet, Jean-Yves Cavaille

**Affiliations:** ^a^ ELyTMaX UMI 3757, CNRS, Université de Lyon, Tohoku University, International Joint Unit, Tohoku University, Sendai, Japan; ^b^ Intelligent Fluid Control Systems Laboratory, Institute of Fluid Science, Tohoku University, Sendai, Japan; ^c^ LGEF EA682, Université de Lyon, INSA-LyonVilleurbanne, France

**Keywords:** Magneto-rheology, energy harvesting, magneto-elastic, composite, 10 Engineering and Structural materials, 206 Energy conversion / transport / storage / recovery, 208 Sensors and actuators

## Abstract

Magneto-rheological (MR) elastomers contain micro-/nano-sized ferromagnetic particles dispersed in a soft elastomer matrix, and their rheological properties (storage and loss moduli) exhibit a significant dependence on the application of a magnetic field (namely MR effect). Conversely, it is reported in this work that this multiphysics coupling is associated with an inverse effect (i.e. the dependence of the magnetic properties on mechanical strain), denoted as the pseudo-Villari effect. MR elastomers based on soft and hard silicone rubber matrices and carbonyl iron particles were fabricated and characterized. The pseudo-Villari effect was experimentally quantified: a shear strain of 50 % induces magnetic induction field variations up to 10 mT on anisotropic MR elastomer samples, when placed in a 0.2 T applied field, which might theoretically lead to potential energy conversion density in the mJ cm^-3^ order of magnitude. In case of anisotropic MR elastomers, the absolute variation of stiffness as a function of applied magnetic field is rather independent of matrix properties. Similarly, the pseudo-Villari effect is found to be independent to the stiffness, thus broadening the adaptability of the materials to sensing and energy harvesting target applications. The potential of the pseudo-Villari effect for energy harvesting applications is finally briefly discussed.

## Graphical abstract

**Figure:**
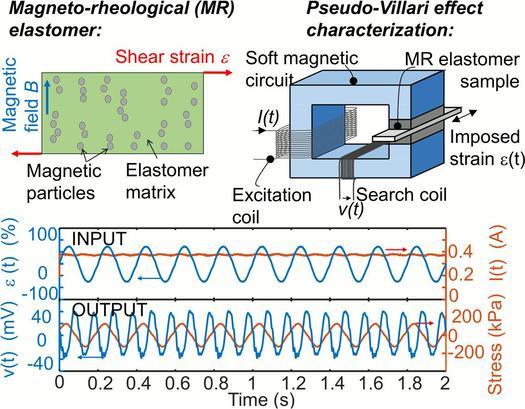

## 1. Introduction

Energy conversion is one of the challenging targets for developing multifunctional materials addressing societal challenges such as energy, transportation and health. A well-known example is piezoelectric materials that convert electricity into mechanical displacement and vice versa. Generally speaking, ‘energy conversion’ covers both (i) actuation where electrical energy aims at producing mechanical displacement (i.e. converse piezoelectric effect, direct electrostriction or magnetostriction) and (ii) sensing and energy harvesting where mechanical energy is used to produce electricity (for instance, from vibrations or periodic motions). In the case of soft materials (i.e. polymers, that are suitable for low-frequency, high-strain devices) electromechanical coupling properties are enhanced, at least at low electrical excitation fields, by the use of heterogeneous materials, where the different phases exhibit strong contrast in their dielectric, magnetic and/or elastic properties. Magneto-rheological (MR) elastomers, as an important branch of the smart material family, are composite materials with magnetically polarized suspended particles or arranged particles, dispersed in a soft matrix (non-magnetic like polydimethylsiloxane [PDMS], rubber, etc.) or even gel matrix with additional ingredients (silicone oil, graphite, etc.) [1–5]. Such a composite exhibits apparent magnetostriction much larger than observed with typical naturally magnetostrictive materials like Terfenol-D [6], which depends on how the particles are distributed within the polymer as well as their aspect ratio. For instance, a randomly filled composite will elongate, whereas a composite with particles distributed in a chain-like structure [7] (by means of a magnetic field applied during crosslinking of the polymer) would contract [8,9]. Moreover, as a consequence of dipole–dipole interactions, the apparent stiffness (Young’s modulus, *E*) is increased by an applied magnetic field too, yielding to the so-called MR effect. Therefore, an external magnetic field can be applied to the MR elastomer to enhance the stiffness and damping properties, with response time in the order of few milliseconds [10]. The MR elastomer’s unique properties make such composites a promising candidate for various applications including adaptive tuned vibration absorbers (ATVAs) [11–14], semi-active base isolators [15,16] and stiffness tunable mounts and suspensions [2,17,18]. In these examples, the role of the magnetic interactions of the particles and their ability to be displaced on a large scale have been highlighted to show the uniqueness of these materials [19]. However, almost all of the applications of MR elastomers consider magnetic to mechanical energy conversion, with no particular emphasis on the bidirectionality of the coupling.

Meanwhile, with the spread of nomad consumer electronics and increasing demands in embedded sensors and sensor networks from industries, the challenge of powering such systems has recently been raised. As an alternative to conventional batteries, the concept of energy harvesting (enabling microelectronic stand-alone systems [20–23]) consists of exploiting energy sources directly available in the environment of the device and converting them into electricity. Among numerous energy sources (e.g. solar, thermal, RF, etc.), vibrations are a premium choice because of their wide availability, high power density and constancy. Conventional small-scale electromechanical conversion, mainly based on the piezoelectric effect, is not well adapted to large-deformation, low-frequency devices because of the rigidity of piezoelectric materials. Also, electrostrictive soft materials require an activation mechanism (high electric field polarization sources) that somehow compromises their true applicability, whereas magnetostrictive and magneto-rheological polymers show low Young’s modulus, and can be easily magnetized through magnets.

Considering both the attractive advantages of MR elastomers and the need for alternative materials for sensing and energy harvesting applications, this work focuses on the capability of MR elastomers to convert mechanical energy into electrical energy via magneto-mechanical mechanisms. By definition, the magneto-mechanical coupling corresponding to a dependence of magnetic induction field on mechanical stress is called the Villari effect [24] (also known as magneto-elastic coupling, or inverse magnetostrictive coupling [25]). In the present study, since an external magnetic field application is required for obtaining such a mechanical to magnetic domain coupling, it is referred to as the pseudo-Villari effect.

MR elastomers were fabricated and tested with a particular focus on their mechanical to magnetic energy conversion potential for sensing and energy harvesting applications. The article is organized as follows: the material fabrication and experimental setup are presented first, followed by thermodynamic considerations allowing the relevant parameters for energy conversion to be identified. The main results of magneto-rheological properties (change of storage shear modulus and loss factor with applied magnetic field) and pseudo-Villari effect (change of magnetic properties with applied shear strain) are then presented, including a discussion of the results. This part is then concluded by an analysis of the results in terms of energy conversion capability for energy harvesting applications.

## 2. Material fabrication and characterization

### 2.1. Materials fabrication

The components for fabricating hard MR elastomers are carbonyl iron particles and silicone rubber; for soft MR elastomers, beside the iron particles and silicone rubber, silicone oil is also involved. Carbonyl iron particles (CIP CS, BASF, Ludwigshafen, Germany) are spherical and 6.0–7.0 μm in diameter. Silicone rubber is synthesized from a base material (KE-1241, Shin-Etsu Chemical Co. Ltd., Tokyo, Japan) and curing agent (CLA-9, Shin-Etsu Chemical Co. Ltd.) at 10:1 weight ratio. Silicone oil (378364, Sigma-Aldrich Co. Ltd., Saint Louis, MO, USA) has a viscosity of 0.0001 m^2^ s^−1^ at room temperature.

**Figure 1.**
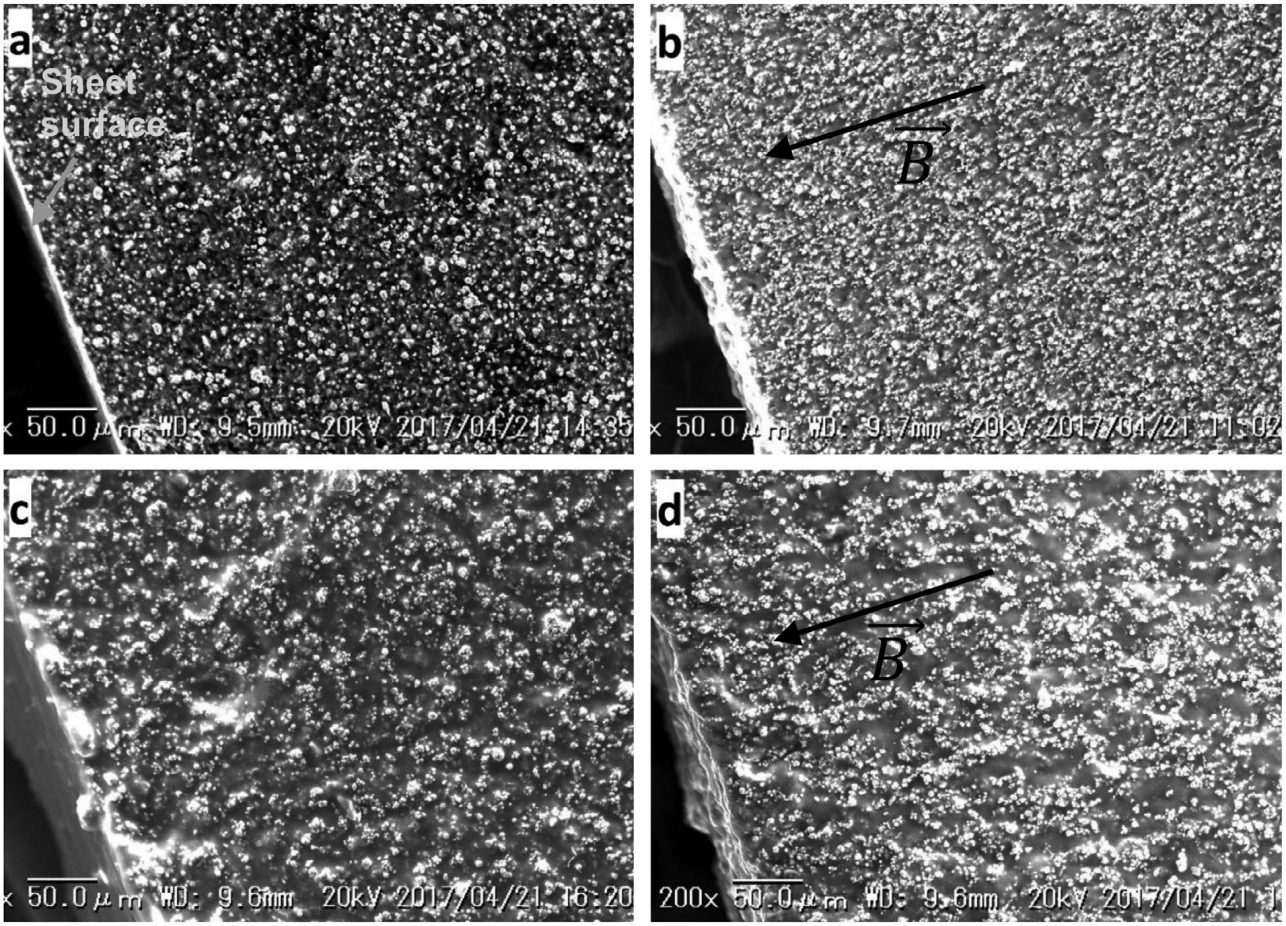
SEM images of MR elastomers: (a) SR_ISO; (b) SR_ANISO; (c), SR_SO_ISO; (d) SR_SO_ANISO. In images (b) and (d), the direction of the applied induction field during curing of the polymer is highlighted by the arrow.

In the fabrication process, carbonyl iron particles, silicone rubber and silicone oil (only for soft MR elastomers for the last-mentioned) at certain concentrations were mixed and stirred sufficiently in a beaker; then the mixture was placed in a vacuum to eliminate air bubbles. The vacuumed mixture was poured into a non-magnetic mold with the internal dimensions of 12 mm in width, 2 mm in depth, and 65 mm in length, to fabricate the rectangular sheet sample of MR elastomer. In order to obtain anisotropic MR elastomers, the mold was placed into a magnetic induction field of 0.3 T in the curing process, perpendicular to the sample surface. Because of the magnetic interaction, dispersed carbonyl iron particles move in the matrix to form chains along the direction of the magnetic field. In case of isotropic MR elastomers, no magnetic field is applied, and the mold was rotated by a servomotor at 60 rpm to avoid particle settling. In the curing process of both anisotropic and isotropic MR elastomers, the mold was heated with a heat gun set to 80 °C for 60 min to accelerate the curing. The components of MR elastomers with various silicone oil and silicone rubber concentrations are summarized in Table 1. A scanning electron microscope (SEM, VE-9800, Keyence, Osaka, Japan) was used to observe the microstructure of the fabricated MR elastomers (Figure 1(a) and (c) for isotropic samples, and Figure 1(b) and (d) for anisotropic samples).

**Table 1.** Composition of MR elastomers (SR_ISO, isotropic hard elastomer; SR_ANISO, anisotropic hard elastomer; SR_SO_ISO, isotropic soft elastomer; SR_SO_ANISO anisotropic soft elastomer).

Samples	Carbonyl iron		Silicone rubber		Silicone oil		B_0_ during crosslinking
	wt%	vol%	wt%	vol%	wt%	vol%	T
SR_ISO	70	26.29	30	73.71	0	0	0
SR_ANISO	70	26.29	30	73.71	0	0	0.3
SR_SO_ISO	70	24.07	15	33.75	15	42.18	0
SR_SO_ANISO	70	24.07	15	33.75	15	42.18	0.3

### 2.2. Experimental characterization setup

**Figure 2.**
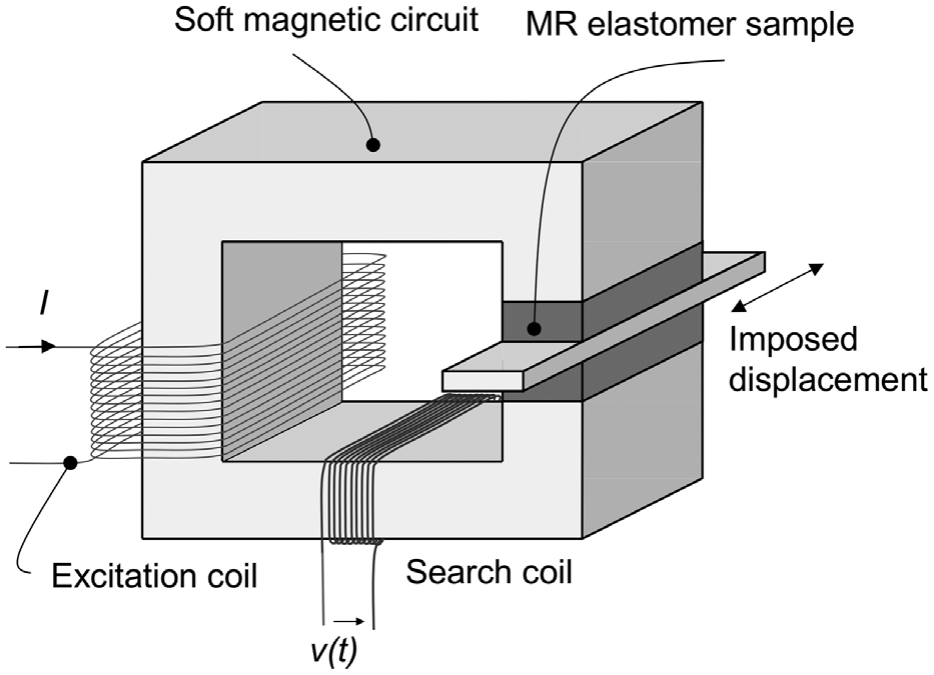
Experimental setup for testing the magneto-rheological materials.

**Figure 3.**
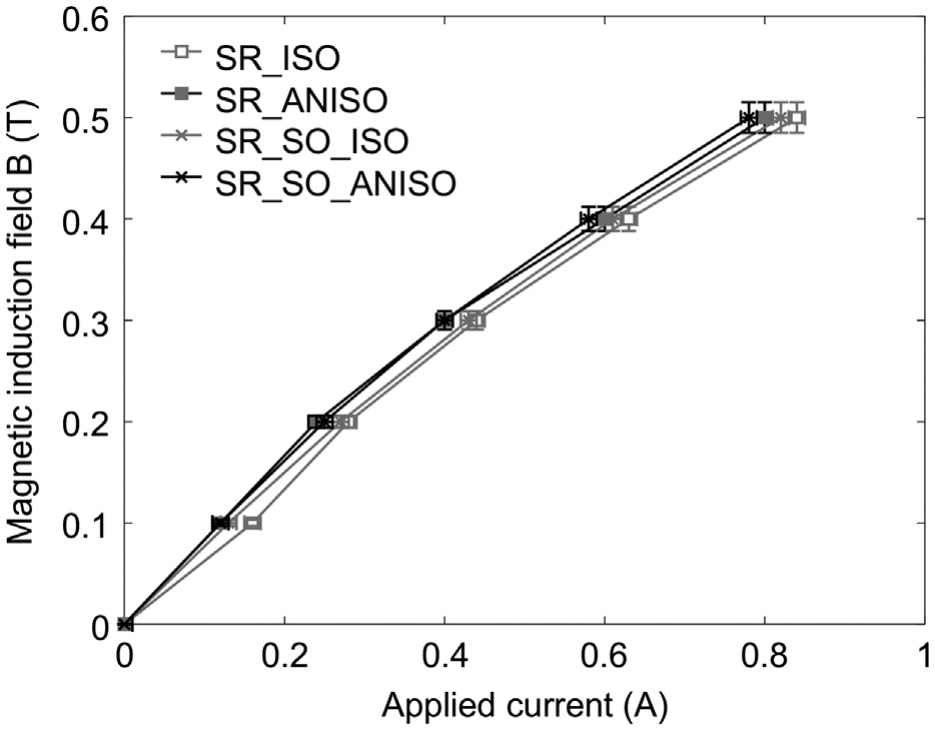
Calibration of the excitation coil used for applying a bias magnetic induction field to the sample.

An experimental characterization setup was developed for measuring both the mechanical property dependence on magnetic field and the dependence of magnetic properties on shear strain (Figure 2). The setup consists of a rectangular-shaped electromagnet featuring an air-gap in order to insert MR elastomer sheets separated by a steel plate. Two pieces of MR elastomers cut to 50 mm in length are installed into two gaps of 2 mm between the two magnetic poles of the electromagnet with the steel plate of 1.0 mm in thickness. The samples are glued by double-sided tape between the surfaces of the magnetic poles and the steel plate to ensure perfect connection. A harmonic displacement is imposed on the steel plate by a slider-crank mechanism and a speed-controlled motor, and the displacement is measured by a laser sensor (LC-2450, Keyence) and a laser displacement meter (LC-2400, Keyence), while the force is measured by a force sensor (LUH-50KF, Kyowa, Tokyo, Japan). The excitation coil is supplied with a constant current (precision ± 0.01 A) for applying a bias magnetic induction field to the MR elastomer sample. The values of obtained magnetic induction fields were determined by a fluxmeter (FM-3001, Denshijiki Industry Co. Ltd., Tokyo, Japan) connected to the search coil prior to sample characterization consisting of integrating the search coil voltage and using step current applied to the excitation coil. The relationship between applied current and magnetic induction field was determined for each sample. On the other hand, in a preliminary experiment, the relation between measured magnetic flux and actual magnetic field in the air gap was determined using a Hall sensor with an overall precision of 3%. For non-strained samples, the calibration is plotted in Figure 3. As expected, the anisotropic samples require lower current for generating a given magnetic induction field thanks to their higher magnetic permeability. However, this calibration is not precise enough to deduce the exact magnetic permeability of the samples, which remains difficult to measure precisely [26]. A tentative estimation from this experimental setup, from the knowledge of the behavior of the steel constituting the magnetic circuit, consists in comparing the reluctance of the magnetic circuit with and without the MR elastomer sample. From this measure, the relative permeability was estimated at 1.6 for both isotropic samples, and 2.0 for both anisotropic samples. The main difficulty in this estimation lies in the soft iron exhibiting remnant magnetization and constituting a non-negligible reluctance in the circuit. The estimated values are, however, consistent with those from the literature, for example with the data of Yin and Sun [27].

The imposed displacement is sinusoidal with constant frequency and amplitude, and the displacement, force and voltage across the search coil are recorded simultaneously. The frequency of the displacement was set to 1 Hz, low enough to avoid any significant skin effect in the magnetic circuit, but high enough to have a reasonable precision on the search coil voltage measurement. Furthermore, such a frequency represents quite well the target applications for the considered device (low-frequency, large-strain energy harvesting, for instance). The dimensions of the experimental setup are given in Table 2.

**Table 2.** Experimental setup main characteristics.

Parameter	Value
MR elastomers dimensions	50 mm × 12 mm × 2 mm
Ferromagnetic circuit total length	20 cm
Ferromagnetic circuit material	Steel SS400
Excitation coil turns	1560
Search coil turns	300

The stress 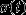 is deduced from force signal 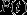 by:(1)
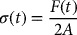



where *A* is the cross-section of the samples (600 mm²) and the shear strain 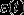 is deduced from displacement 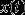 using:(2)
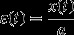



where *a* is the sample thickness (2 mm for all experiments).

From these data, the complex shear modulus components *G* = *G*′ + *jG*′′ = *G*_0_ *e* ^*jδ*^, were determined (*G*′, *G*′′ and *δ* being storage shear modulus, loss shear modulus and loss angle, respectively), with a particular emphasis on the storage modulus *G*′ and loss factor (or internal friction coefficient) *tan δ*.

Magnetic induction field variations, Δ*B*, are deduced from the voltage of the search coil *v(t)* as:(3)
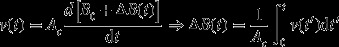



with *A*_*c*_, *B*_0_ and *B(t)* the area of circuit, the applied bias magnetic induction field and the magnetic induction versus time, respectively.

**Figure 4.**
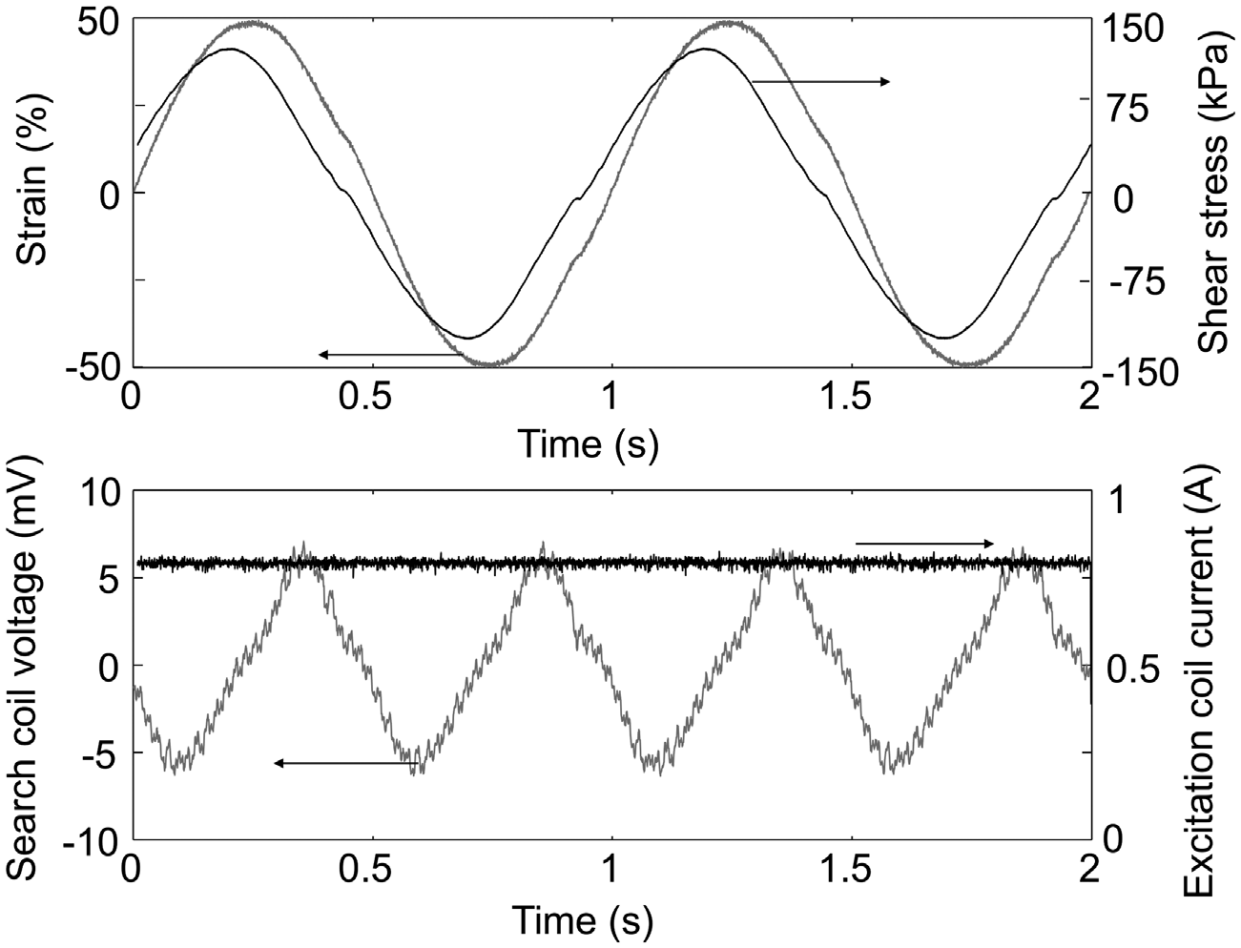
Typical experimental signals of shear strain and stress, the voltage on the search coil and current in the excitation coil. Depicted results are obtained from anisotropic MR elastomer (SR_ANISO) under a magnetic field of 0.5 T.

Figure 4 shows characteristic time signals. A sequence of 20 cycles of shear strain were applied to the sample. The behavior was found to be reproducible over all cycles. The two last cycles are displayed in the figure for better readability. It is noteworthy that the search coil voltage signal features a doubled frequency compared to the strain signal, which is consistent with the expected symmetry of the system (positive or negative strains have the same effect on the magnetic properties) and quadratic behavior of the pseudo-Villari effect.

**Figure 5.**
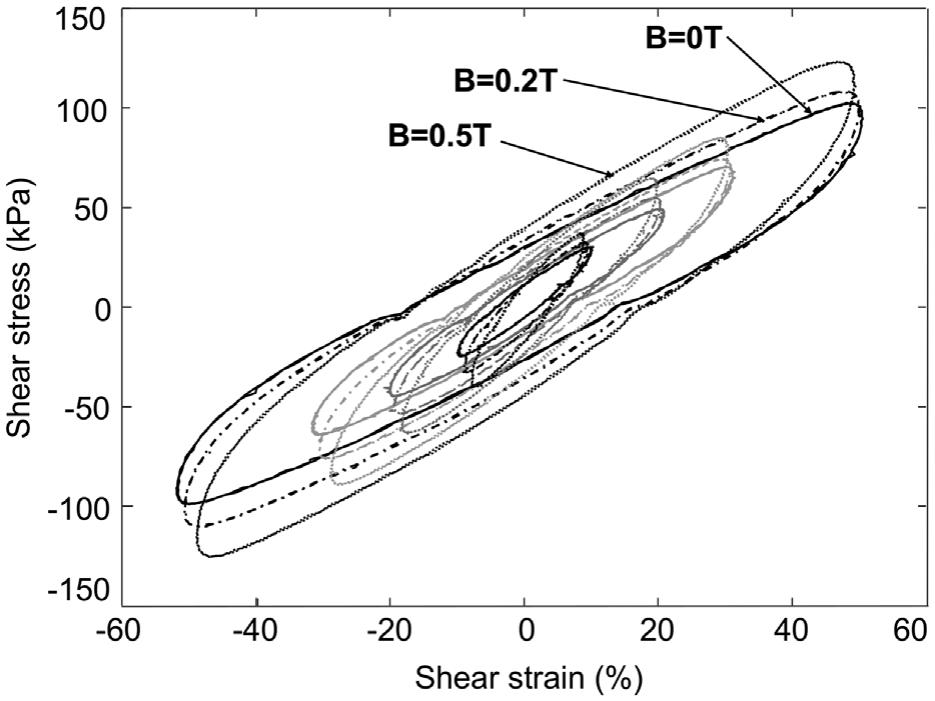
Typical stress-strain curves for bias magnetic induction fields ranging from 0 T to 0.5 T in an anisotropic MR elastomer (SR_ANISO sample).

The mechanical stress-strain curve was then determined for each tested static magnetic field applied through the excitation coil, and an example of the results is displayed in Figure 5. When increasing the magnetic field, the increase of maximum stress is visible on the results, as well as a slight increase of cycle area, thus denoting an increase of mechanical losses too.

## 3. Thermodynamic considerations

The relationship between magnetic and mechanical quantities may be assessed using thermodynamic potential derivation. It could even be possible to obtain the polynomial constitutive equations of the complete magnetic and mechanical behavior in principle. Several assumptions are, however, required, such as the material ergodicity and behavior free of frequency dependence, creep effects or hysteresis. As detailed in the experimental results section, the previous assumptions are questionable, but the thermodynamic analysis is still of interest for showing at least qualitatively the trends of the magneto-elastic coupling.

The material state is determined by two state magnetic variables (magnetic excitation field *H* and magnetic induction field *B*), two mechanical state variables (mechanical shear stress *τ* and mechanical shear strain *ε*) and two thermal quantities (temperature *T* and specific entropy *S*). In the following, independent variables are chosen to be *B* and *ε*, although similar analysis can be performed using other independent variables [28]. Given the enthalpy potential *F* = *U*-*TS*, where *U* is the internal energy, state variable variations at constant temperature are given by(4)
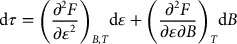

(5)
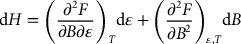



Using the following coefficients (all are functions of 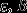 in the general case, without any loss of generality):(6)
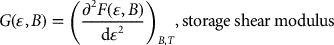

(7)
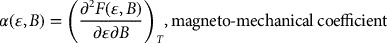

(8)
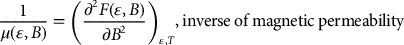



the constitutive equations yield(9)
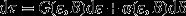

(10)
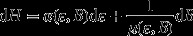



The magneto-mechanical coefficient is then defined as:(11)
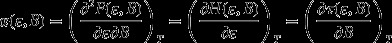



This coefficient is responsible for the shear stress variations when a magnetic induction field is applied and is therefore directly related to the quantification of the magneto-rheological effect. The same coefficient then drives the changes in magnetic properties when a strain is applied. It is therefore expected that materials exhibiting a large magneto-rheological effect should also exhibit a large pseudo-Villari effect. Moreover, it is shown in the experimental part of this study that the tendencies are qualitatively similar. For simplicity of presentation and interpretation of experimental results, we define the pseudo-Villari coefficient as the derivative of the magnetic induction field by the strain at constant excitation field. From Eq. (10), the magnetic induction variation is given by(12)
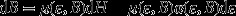



yielding,(13)
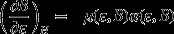



## 4. Results and discussion

### 4.1. Characterization of magneto-rheological properties

Stress-strain curves were approximated by the dynamic viscoelastic behavior, whose storage modulus *G*′, loss modulus *G*′′ and loss factor (*tan δ* = *G*′′*/G*′) were fitted to the experimental data (least-squares solution of an elliptical fit to the measured curve). Considering that the shear strain and stress are written as 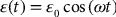 and 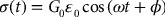, and the complex shear modulus as *G* = *G*′ + *jG*′′, strain and stress are solutions of the following elliptic equation:(14)
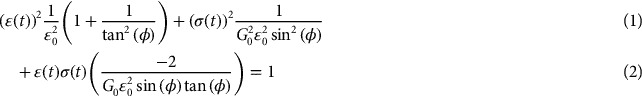



Matlab® least-squares solver is used for determining the best set of coefficients to minimize the error, each time step providing one point in the *ε*(t) / σ(t) plane. Values of strain amplitude and complex shear modulus are then deduced from fitted ellipse coefficients. Moreover, for each fitted curve, the estimated standard errors of the fitting parameters are determined by the solver. A large error bar means that either the signals were noisy, or the shape of the stress-strain curve differs significantly from an ellipse. For most of the tested materials and conditions, the standard error remains lower than 5% for both *G*′ and *tan δ*.

**Figure 6.**
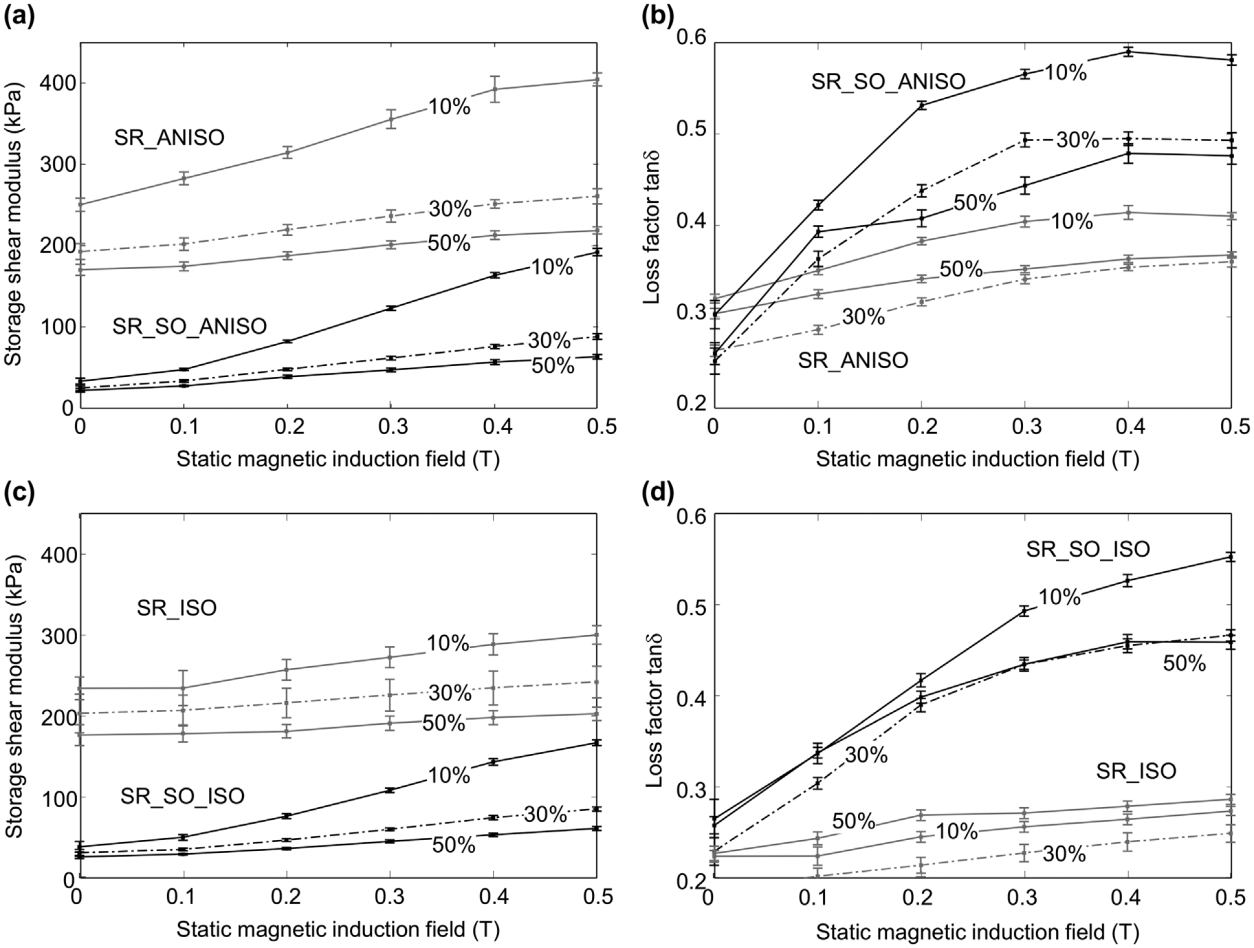
Storage shear modulus and loss factor (*tan δ*) for the four tested materials. (a) Storage shear modulus as a function of induction field for hard and soft anisotropic samples, (b) loss factor *tan δ* as a function of induction field for hard and soft anisotropic samples, (c) storage shear modulus as a function of induction field for hard and soft isotropic samples, (d) loss factor *tan δ* as a function of induction field for hard and soft isotropic samples. For all plots, the shear strain amplitude is indicated in each line.

The results for the four tested materials are displayed in Figure 6. Figure 6(a) shows the storage shear modulus as a function of static magnetic field, for strain levels of 10%, 30% and 50%, in the case of soft and hard anisotropic MR elastomers, whereas the soft and hard isotropic sample behaviors are displayed in Figure 6(c). Figure 6(b) shows the loss factor *tan δ* for the same conditions (magnetic field and strain levels) in the case of soft and hard anisotropic MR elastomers, whereas the soft and hard isotropic sample loss behaviors are displayed in Figure 6(d). The tendencies are consistent with already published data on MR elastomers with similar weight fraction (70%) of iron particles [4]. It can be seen that all MR elastomers show very distinct storage modulus *G*′ increase while increasing the applied magnetic flux density *B*, which is the so-called MR effect, as unique properties of MR elastomers. Compared with isotropic MR elastomers, anisotropic samples have a larger relative increase of storage modulus, which has been observed in previous works [29]. The absolute values of the increase in storage modulus from 0 T to 0.5 T for both hard and soft anisotropic MR elastomers are very close.

As detailed in Table 3, for a strain of 10%, Δ*G*′ ~ 160 kPa in the case of soft anisotropic elastomers, whereas Δ*G*′ ~ 154 kPa in the case of hard MR elastomers. It should be noted that the relative variation is very close for all tested strain amplitudes. This shows that the addition of silicone oil does not affect the storage modulus increase in the anisotropic MR elastomers. It is also noted that the soft MR elastomers’ zero-field storage modulus (*G*_0_′ = 34 kPa for 10% strain) is lower than that of the hard MR elastomers (*G*_0_′ = 250 kPa), roughly eight times less; this means that the addition of silicone oil not surprisingly reduces the initial stiffness of MR elastomers. However, the maximum storage modulus relative increase, i.e. Δ*G*′*/ G*_0_′, of the hard MR elastomers is lower than that of the soft MR elastomers because hard MR elastomers have larger zero-field storage modulus *G*_0_′ (see Table 3 for experimental values).

**Table 3.** Relative and absolute variations of storage shear modulus in soft and hard MR elastomers. Absolute storage shear modulus is defined as Δ*G*′ = *G*′*(B* = 0*.*5 *T)* – *G*′*(B* = 0 *T).* Relative storage shear modulus variation is defined as Δ*G*′*/G*_0_′, where *G*_0_′ = *G*′ = *G*′*(B* = 0 *T)*.

Material	Strain level	*G*_0_′ (kPa)	Δ*G*′ (kPa)	Δ*G*′*/ G*_0_′
SR_SO_ANISO	10%	34	160	470%
	30%	25	63	250%
	50%	22	41	180%
SR_ANISO	10%	250	154	60%
	30%	193	68	35%
	50%	170	48	28%

The applied shear strain amplitude also influences the storage modulus [30]. For each MR elastomer sample, when the applied shear strain amplitude increases, the storage modulus shows a lower value; meanwhile, the storage modulus increasing rate also decreases with increasing shear strain amplitude. For each MR elastomer, the loss factor *tan δ* also shows an increasing trend when raising the magnetic flux density *B*, although the relationship between the loss factor and the shear strain amplitude requires further investigation. In addition, the interpretation of the increase of *tan δ* with increasing magnetic field is far from obvious, as mechanical losses in soft composite materials have many different origins. Without an applied magnetic field, both composites (soft and hard matrix based MR elastomers) exhibit quite similar behaviors in terms of loss factor. The anisotropic samples’ loss factor at *B* = 0 T ranges between 0.23 and 0.32 (depending on strain level), whereas isotropic samples’ loss factor ranges between 0.18 and 0.26. These values look close to each other considering the huge increase of the loss factor with the magnetic induction field (up to 0.6 as shown in Figure 6(d)). The main difference between soft and hard composites is more evident on the real part of the complex modulus: the softer matrix was obtained by the dissolution of a viscous solvent (silicon oil acting as a plasticizer) within the polymer before its polymerization. Under zero magnetic field, these composite materials act like all similar composites made of a soft matrix embedding randomly dispersed stiff particles. It is known that interface areas between particles and matrix are places where dissipations are enhanced by different phenomena: de-adhesion/adhesion of macromolecules at the particles surface (Payne effect) [31] and local damage at high strain (Mullins effect) [32], etc. For more complete insight, see for instance Muhr [33]. One of the reasons comes from the heterogeneous stress-strain fields induced by the fillers. More precisely, when a magnetic field is applied, a new solicitation is superimposed due to aggregates of particles formed during the material processing step that are locally anisotropic and submitted to complex magnetic force fields. The stress transfer occurs now from the particles to the matrix, which might lead to strong and highly localized deformations, which in turn might be the origin of these observed high losses. On the other hand, the stiffer the material, the smaller the deformation and in turn the energy loss. Whatever the case may be, such analysis would require more observations, more specifically microstructural characterization, which is ongoing work and beyond the scope of the present study.

It can be concluded that the four tested MR elastomers exhibit similar MR effect when considering the absolute variation of shear storage modulus Δ*G*′ as a function of magnetic induction field (variations of modulus ranging from 40 kPa to 160 kPa depending on strain level). Consequently, the relative variations are much higher for soft MR elastomers than for hard elastomers. In addition, loss factor variations are much larger for soft elastomer matrix (from 0.2 to 0.6) compared with hard elastomer matrix (from 0.2 to 0.35).

### 4.2. Characterization of the pseudo-Villari effect

**Figure 7.**
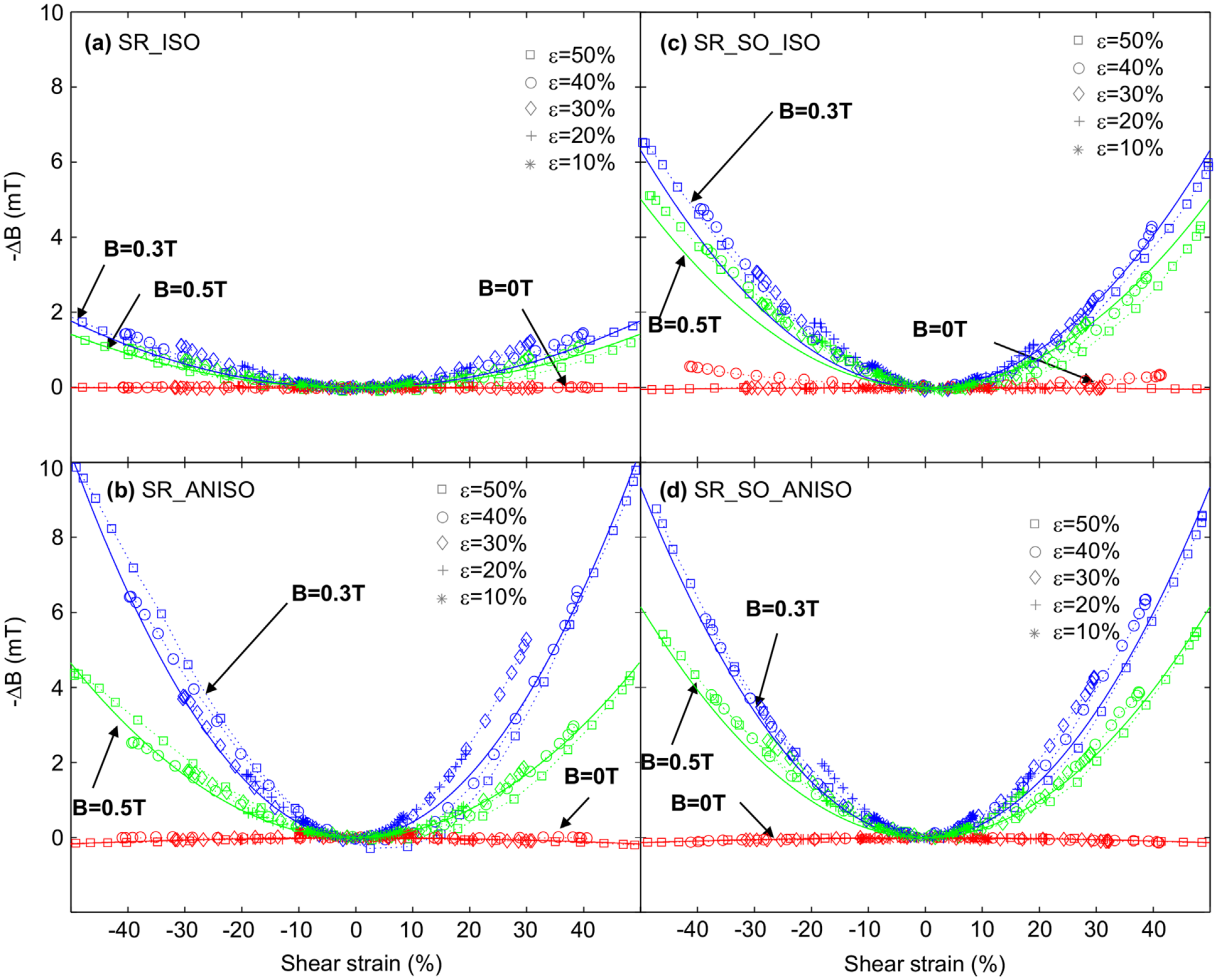
Magnetic induction field variations versus shear strain on the different MR elastomer samples. Magnetic induction field is deduced from search coil voltage, and the shear strain was harmonic with a frequency of 1 Hz. The solid line is a quadratic fit to experimental data. Blue color is for a bias magnetic field of 0.3 T, green color for 0.5 T and red color for 0 T. Different symbols are used for the different strain amplitude levels as shown in the legend of each subfigure.

From the voltage measurements, the magnetic induction field variations were calculated by integrating the voltage of the search coil (Equation (3)). A time-dependent signal, which can be compared to both shear stress and strain signals, is thus obtained. In order to better understand the link between magnetic properties and shear strain, the resulting magnetic induction field variations are plotted against shear strain (Figure 7). The variations of magnetic induction field are plotted as positive variations for the sake of clarity (although being negative since ∂H/∂*ε* = ∂τ/∂B > 0 and μ > 0 so ∂B/∂*ε* = − μ ∂H/∂*ε* < 0).

In Figure 7, three different bias magnetic fields are considered: 0 T, 0.3 T and 0.5 T respectively. In each graph, the different tested shear strain amplitudes are superimposed to each other. It is noteworthy that the different curves overlap reasonably and follow more or less a quadratic behavior with the shear strain. Comparing isotropic (Figure 7(a) and (c)) and anisotropic materials (Figure 7(b) and (d)), it is evidenced that the effect is much larger in the case of anisotropic material, reaching variations up to Δ*B* ~ 10 mT for a strain of 50% and bias magnetic flux density *B*_0_ ~ 0.3 T.

**Figure 8.**
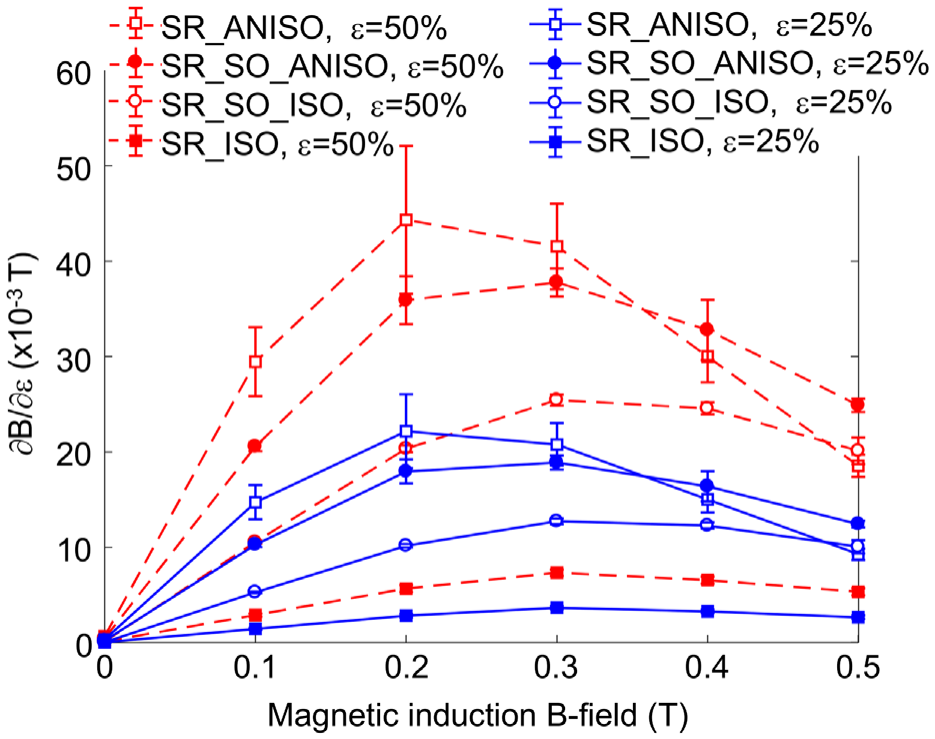
Pseudo-Villari coefficient as a function of bias magnetic induction field for a strain level of 25% and 50% for the four tested materials.

The quadratic behavior described above was fitted to a second-order polynomial expression using Matlab® least squares solver giving both the fitted parameter and the standard deviation. The magnetic induction field being written as *B* = *B*_0_ + *aɛ* ^2^, the pseudo-Villari coefficient is calculated directly by deriving the magnetic induction field: 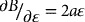. It is calculated for a shear strain of *ε* = 50% and *ε* = 25%, for all bias magnetic induction fields and all tested materials. The relative standard deviation of the plotted parameter 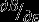 and of the fitted parameter *a* are identical, and all results are displayed in Figure 8. Interestingly, the maximum magnetic induction variations are observed for an intermediate magnetic field of 0.3 T. It is noteworthy that both hard and soft elastomer-based anisotropic samples exhibit rather similar behavior, with a coefficient reaching 44 × 10^−3^ T and 38 × 10^−3^ T, respectively, for a strain of 50%. Recalling the magneto-rheological properties, the variation of the storage modulus with induction field was of the same order of magnitude for both materials (i.e. for Δ*B* = 0.5 T, in the case of a hard anisotropic MR elastomer, Δ*G*′ ~ 48 kPa at strain of 50%, whereas Δ*G*′ ~ 41 kPa for a soft one, see Table 3). It is then consistent with the observation here concerning the pseudo-Villari effect, since the absolute variations of storage modulus have more significance than relative variations. The origin of the MR effect indeed lies in the particle–particle interaction inducing an additional stress of magnetic origin (corresponding to *ε*Δ*G*′), which is independent on the matrix initial stiffness. Similarly, the pseudo-Villari effect is related to the particle displacement induced by the shear strain. In case of hard and soft MR elastomers, the particle distribution and alignment – or at least the way the distribution depends on applied shear strain – should be similar for both materials. Moreover, the pseudo-Villari effect shown in Figure 8 passes through a maximum and then decreases significantly for both anisotropic and isotropic elastomers. However, the anisotropic elastomer maximum is reached for an applied induction of 0.2–0.3 T, while isotropic elastomers maximum is reached around 0.3–0.4 T. The origin of such a maximum might be related to the capability of the particles in realigning themselves under high magnetic induction despite the imposed shear strain. This results in the particle distribution being similar for strained and non-strained states, leading to a lower pseudo-Villari coefficient. The mobility of the particles in the matrix, or their capability to further stress the matrix locally, is nevertheless difficult to consider quantitatively.

As detailed through thermodynamic considerations (section 3), the pseudo-Villari coefficient is related both to the magneto-rheological effect and to the change in magnetic induction field when a shear strain is applied (i.e. Eq. (11) showing the cross-derivatives of the enthalpy *F*, and the equivalence between both effects). From the results presented in Figure 6, the coefficient should be roughly the slope of the curve 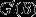. The maximum value of the slope is also obtained for bias magnetic fields of around 0.2–0.3 T, thus showing that the maximum magneto-rheological effect occurs under the same conditions as the maximum pseudo-Villari effect. However, further quantification of this relation is tricky because of the difficult determination of the thermodynamic stress from experimental results (i.e. retrieving the stress linked to enthalpy variations and not to hysteresis or losses).

As discussed above, anisotropic and isotropic samples have the same pseudo-Villari effect sign, but a slightly larger effect is observed for anisotropic samples. However, considering magnetostriction (being also a magneto-mechanical coupling), the change of length (longitudinal mode) has a different sign for isotropic and anisotropic MR elastomers [8,34,35], and the maximum magnetostrictive activity is observed in isotropic MR elastomer [36]. This difference on the effect of particle alignment on those two different magneto-mechanical couplings is probably related to the longitudinal mode for magnetostriction and shear more for the pseudo-Villari effect. In the case of the magneto-rheological effect in compression mode, Varga et al. [37] investigated the effect of anisotropy on magneto-rheological properties. They performed stress-strain tests along and perpendicularly to the chain structure while applying an external field with different direction relative to the particles structures. As with the results presented here, they observed that the maximum magneto-rheological effect is obtained in anisotropic materials.

Regarding the pseudo-Villari effect itself in MR elastomers, Russkikh [38] studied the Villari effect (also called magneto-elastic effect) on magneto-rheological elastomers in compression mode. In the Russkikh study, the magnetic field variations induced by a compression strain were measured. No external magnetic field was applied during the test, the magnetic particles exhibiting remnant magnetization. The measured magnetic field variations were due to the change of interaction between the particles that modified their magnetic state. For strains up to 25% in compression, a change was reported in the magnetic induction (also following a quadratic dependence on the compression strain) of up to 0.3 mT, although the application of an external magnetic field source was not considered. In this case, the measured effect is a true Villari effect, where magnetic field variations occur in the absence of an external excitation field.

In a comprehensive investigation of magnetostriction and field stiffening of MR elastomers, Han et al. [28] pointed out both theoretically and experimentally the magnetic permeability dependence on mechanical strain. It was especially highlighted that the susceptibility variations with applied strain are quadratic, similarly to this study. Without bias magnetic field, they obtained magnetic susceptibility variations up to 8% for a 16% compressive strain level on an anisotropic MR elastomer sample with 40 wt% magnetic particles. In their model and thermodynamic potential writing, they especially linked the stress equation to the magnetic equation through a common parameter validated by experiments with a rather convincing precision. In the present work, the close relation between MR effect and pseudo-Villari effect was also shown as detailed previously.

It can be concluded that (i) the pseudo-Villari effect is large in shear mode, (ii) anisotropic MR elastomers exhibit better coupling than isotropic ones, and (iii) the effect has little dependence on the properties of the embedding matrix which can be softened without any reduction in the pseudo-Villari effect. This last-mentioned result is of great importance in terms of energy conversion.

### 4.3. Application to energy harvesting

In the previous section we demonstrated the dependency of the magnetic induction field on the shear strain at constant excitation field, with variations up to 10 mT. This section investigates how this property may lead to effective electromechanical energy conversion. As it is intended to give energy density in the view of a material’s properties, no specific device will be considered, using preferably theoretical thermodynamic energy conversion cycles. For the sake of simplicity, we consider here only the purely magneto-mechanical conversion, and the further electromagnetic system is not taken into account here. This simplification is of course much questionable, but the design of a magnetic circuit and associated coils is beyond the scope of this work.

**Figure 9.**
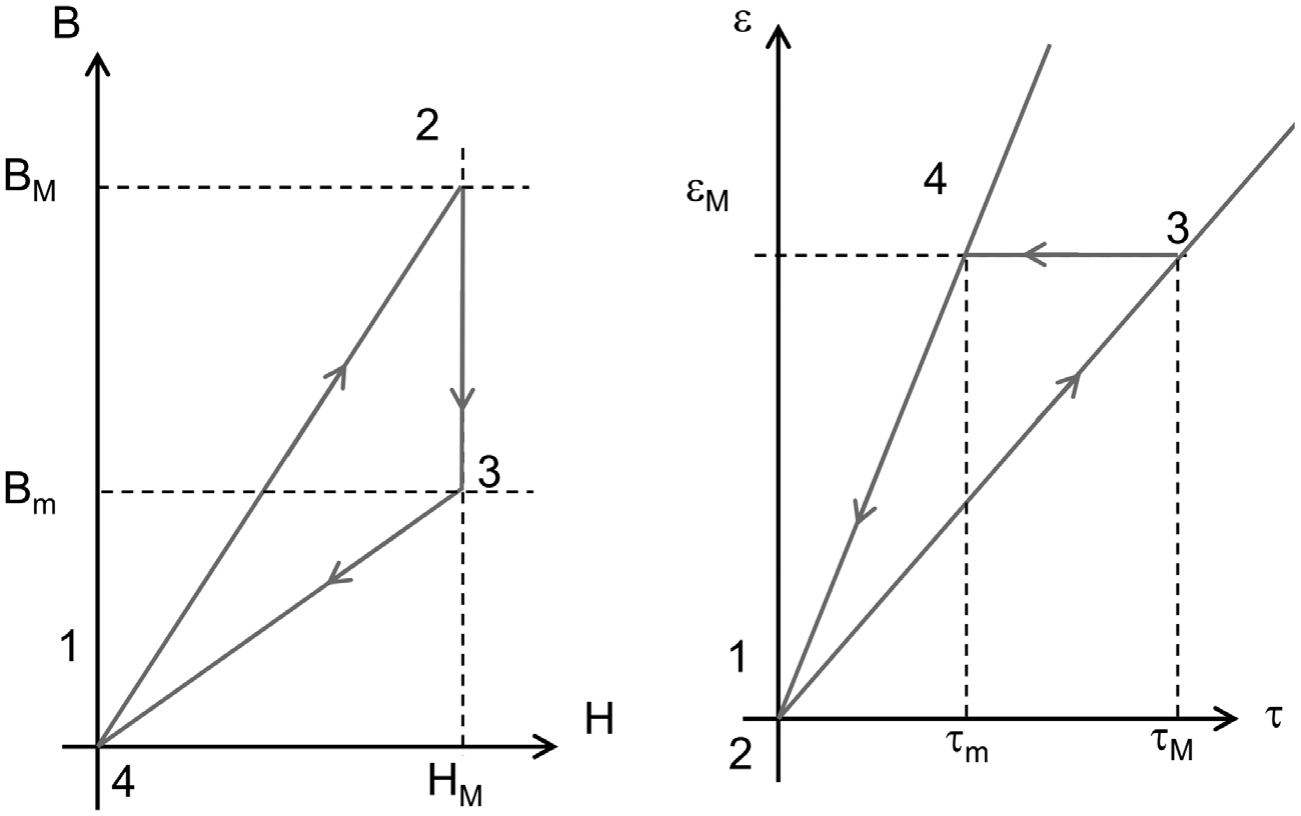
Thermodynamic cycles used for the estimation of energy conversion capability.

The first step lies in considering the following energy conversion cycle (equivalent to an Ericsson cycle by replacing thermal quantities by magnetic quantities) illustrated in Figure 9. During the first step (1–2), the magnetic excitation field is increased to a value *H*_*M*_ at zero shear strain. During the second step (2–3), the excitation field *H*_*M*_ is kept constant and the material is strained up to a strain of *ε*_*M*_. This results in a decrease of magnetic induction field as measured and displayed in Figure 7. During the third step (3–4) this excitation field is decreased to zero, and shear strain is decreased to zero during the last step (4–1). The closed loop area in the *B*/*H* diagram corresponds to the energy which is converted from mechanical operator to a magnetic energy. Its value is given by:(12)
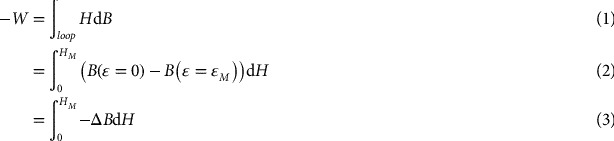



**Figure 10.**
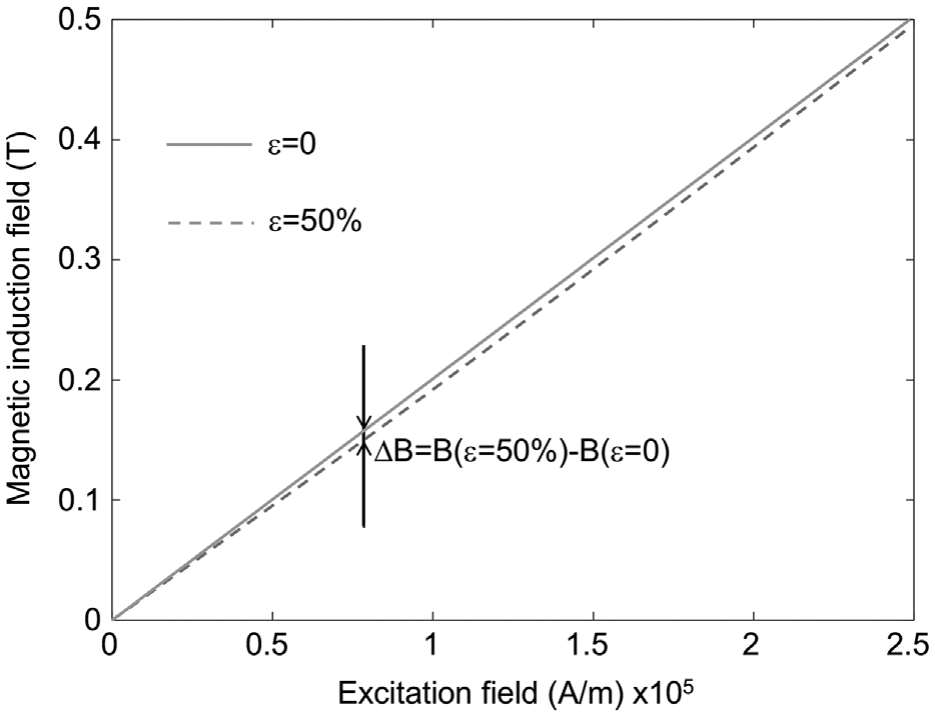
Magnetic behavior of SR_ANISO MR elastomer under zero shear strain and 50% shear strain conditions.

Typical *B* versus *H* behavior is illustrated in Figure 10 in the case of an anisotropic hard matrix elastomer (SR_ANISO). The zero-strain magnetic behavior is assumed to be linear for the sake of simplicity. It should be noted that since the energy is the integral of the magnetic induction variation, it does not depend on the behavior of *B* versus *H* at zero strain. The curve at a shear strain of 50% is obtained by subtracting from the previous curve the values of magnetic induction field variations.

**Figure 11.**
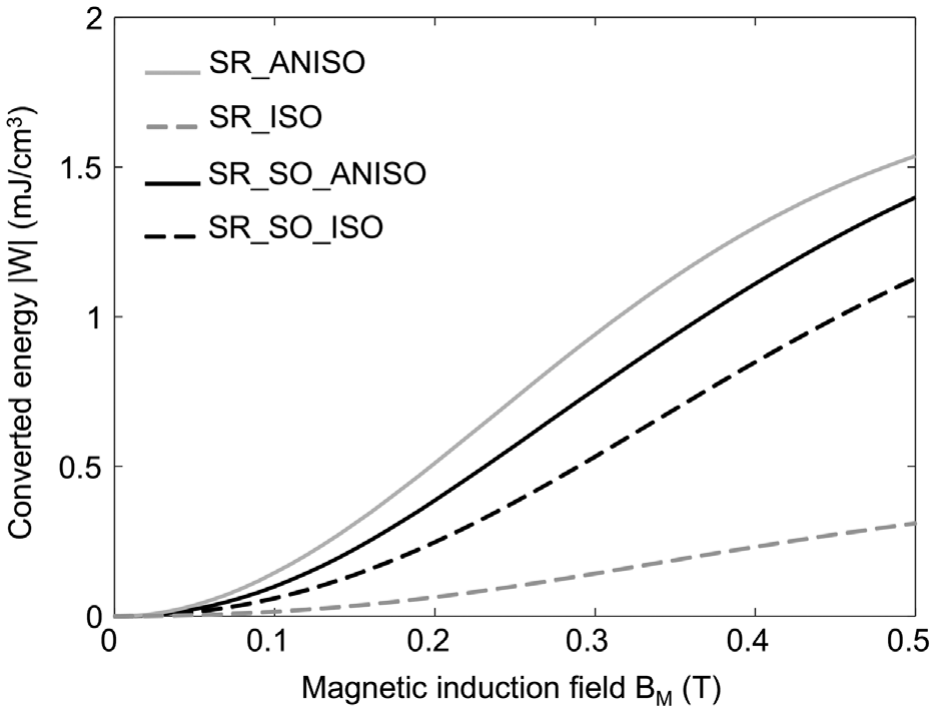
Energy conversion for the different MR elastomers for a strain of 50%, as a function of the maximum magnetic induction field *B*_*M*_ applied during the corresponding thermodynamic cycle.

The relation between *H*_*M*_ and magnetic induction field *B*_*M*_ for each sample was estimated by considering a relative permeability of 2 for anisotropic samples and 1.6 for isotropic samples. The actual magnetic permeability of the tested samples is an estimation only as detailed in the experimental setup section. Although imprecise, this still allows a reasonable estimation of the order of magnitude of the converted energy, which is roughly proportional to the permeability and to the magneto-mechanical coupling coefficient. Converted energy estimation is displayed for the four tested MR elastomers in Figure 11.

When increasing the maximum magnetic induction field of the energy conversion cycle, the converted energy increases, and the slope decreases from around 0.3 T, which is consistent with the decrease of the pseudo-Villari coefficient. Anisotropic samples show the highest converted energy, up to 1.5 mJ cm^−3^ for a maximum strain of 50% and a maximum magnetic induction field of 0.5 T in the case of SR_ANISO. Such an energy density is comparable to those typically obtained in an electromechanical system featuring other coupling effects [39]. It is interesting to compare this value to the stored mechanical energy when straining the material. Based on the storage shear modulus values, this represents around 40 mJ cm^−3^ in the case of hard elastomer matrix (SR_ANISO), and around 12 mJ cm^−3^ in the case of the soft matrix (SR_SO_ANISO). Similarly to piezoelectric materials, a magneto-mechanical coupling factor may be defined as the square root of the ratio between the converted energy and the total stored mechanical energy at the maximum strain state.(13)
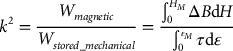



Based on the results presented here, the converted energy might reach up to 12.5% of the total stored mechanical energy, corresponding to a coupling factor *k* of 35%, almost half of the best piezoelectric materials [40]. The advantage of soft MR elastomer is, however, counter-balanced by a higher level of mechanical losses (around 40%), which leads in turn to a low efficiency of the energy conversion. Further considerations related to energy harvesting would require the development of preliminary proof of concept, where the magneto-electrical energy conversion has to be designed. However, as recalled above, MR elastomers are particularly suitable to low-frequency, large-strain energy harvesting (unlike piezoelectric materials), and magnetization is easier to achieve (using magnets) than electrical polarization in electrostrictive materials.

Hence, from the energy conversion from the mechanical domain to the magnetic one, a further step can be included to convert this magnetic energy into electrical energy, for instance using a coil exploiting the Lenz effect allowing the appearance of a voltage *V*_*lenz*_ due to the change of the magnetic induction field (similarly to Eq. (3), where the effective area *A* can be increased by appropriate design of the coil, e.g. cross-section and number of turns).

While the previous demonstration effectively showed the possibility of harvesting energy with the proposed materials, implementing energy harvesting in a realistic fashion would necessitate the removal of an external control. Although beyond the scope of this paper, a preliminary possibility to obtain a realistic system would rely on permanent magnets for magnetization purposes, hence shaping the energy cycle close to an ellipsoid.

## 5. Conclusions

MR elastomers are barely investigated for mechanical to magnetic/electrical energy conversion. In this work, we investigated both the MR effect (dependence of mechanical properties on a magnetic field) and the pseudo-Villari effect (dependence of magnetic field on mechanical strain). Anisotropic and isotropic magneto-rheological elastomers based on soft and hard elastomer matrices exhibit a significant variation of their mechanical properties upon the application of a magnetic induction field. Relative variations of 30% in the case of hard elastomer and more than 200% in the case of soft elastomer were observed. The pseudo-Villari effect was experimentally evidenced through magnetic flux variations as a result of the application of the shear strain under constant excitation magnetic field. Isotropic soft and hard elastomers exhibit different behavior, soft elastomers showing large magneto-rheological and pseudo-Villari effects, whereas hard elastomers do not. Interestingly, anisotropic soft and hard elastomers exhibit rather similar behavior both for their magneto-rheological effect (Δ*G*′ = 48 kPa and Δ*G*′ = 41 kPa, respectively, for an induction field increasing from 0 to 0.5 T) and for their pseudo-Villari effect (Δ*B* = 10.2 mT and 9.4 mT, respectively, for 50% strain and bias magnetic field of 0.3 T). Based on this observation, it can be concluded that the magneto-elastic coupling (including both magneto-rheological and pseudo-Villari effects) in anisotropic MR elastomers depends on the particle type and volume fraction only, probably because it originates solely from the dipole–dipole and induction field–dipole interactions. As a consequence, the initial properties of the matrix can be tailored with respect to the application, for controlling shear modulus and loss factor for example, without any loss of performance in terms of coupling between magnetic and mechanical energies. However, the loss factor is found to be much higher for soft MR elastomers, and we should investigate if the process of adding silicon oil for softening the matrix does not induce at the particle–matrix interface a lack of adhesion due to the accumulation of plasticizer (silicon oil). New tests should be performed on materials without additives, but less crosslinked to avoid the presence of an additional phase.

In terms of energy harvesting applications, the densities of energy conversion potential were estimated at 1.5 mJ cm^−3^ using theoretical energy conversion cycles (induction field of 0.5 T and shear strain of 50%). Further work should be devoted to the development of proof-of-concept electromagnetic energy conversion devices and to detailed investigations of the physical mechanisms of the pseudo-Villari effect for improving the material design.

## Funding

This work was partly supported by the JSPS Core-to-Core Program, A. Advanced Research Networks `International research core on smart layered materials and structures for energy saving’; French Auvergne-Rhone-Alpes; Institute of Fluid Science, Tohoku University, General Collaborative Program [grant number J16067]; French Agence Nationale de la Recherche, Investissements d’Avenir, Programme Avenir Lyon Saint-Etienne de l’Université de Lyon [grant number ANR-11-IDEX-0007].

## Disclosure statement

No potential conflict of interest was reported by the authors.
